# Supplementary figures and images for: Prenatal Alcohol Exposure Disrupts CXCL16 Expression in Rat Hippocampus: Temporal and Sex Differences
Source: Int J Mol Sci. 2025 Feb 23;26(5):1920. doi: 10.3390/ijms26051920 (PMC11900973; doi:10.3390/ijms26051920)

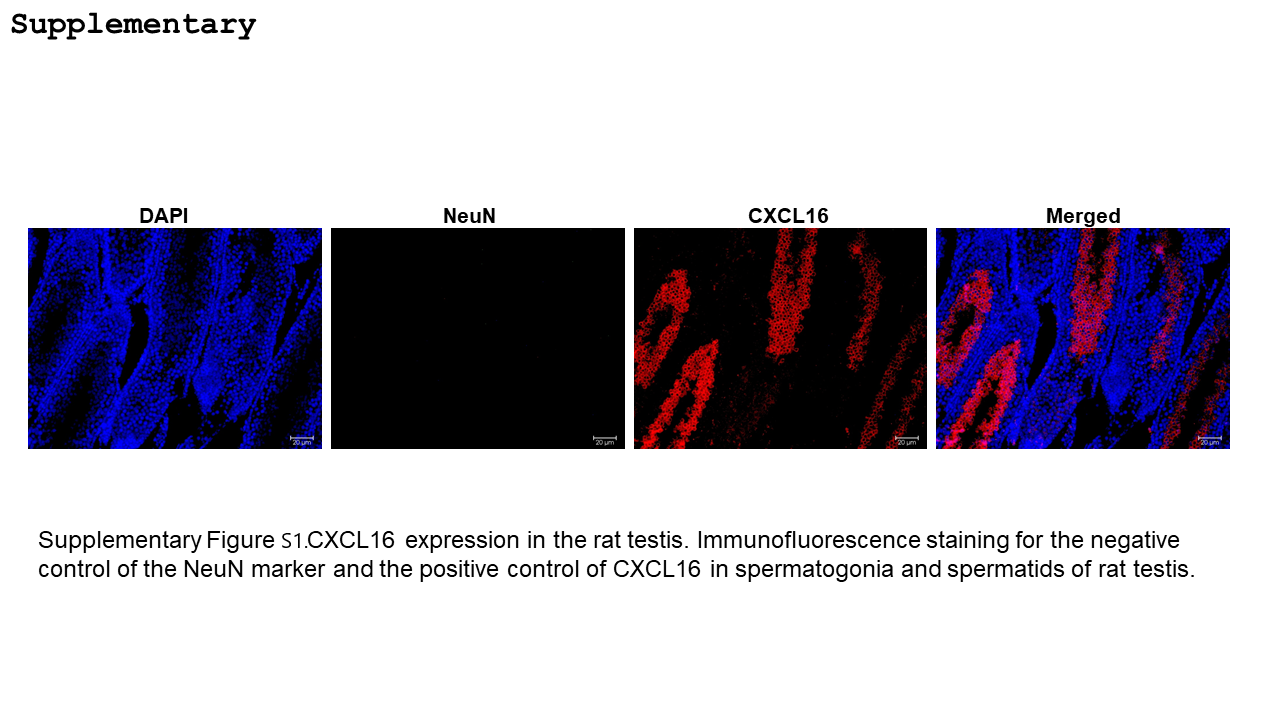

Supplement: Supplementary file 1 [file ijms-26-01920-s001.zip › ijms-3463635-supplementary.tif]
